# Occupational skin diseases from 1997 to 2004 at the Department of Dermatology, University Hospital of Northern Norway (UNN): an investigation into the course and treatment of occupational skin disease 10–15 years after first consultations with a dermatologist

**DOI:** 10.3402/ijch.v75.30100

**Published:** 2016-05-10

**Authors:** Rosemarie Braun, Lars Kåre Dotterud

**Affiliations:** 1 Department of Dermatology, University Hospital, Tromsø, Norway; 2 University of Tromsø, Northern Norway, Tromsø, Norway

**Keywords:** dermatitis, occupational, dermatitis, contact, hand eczema, quality of life, sick leave/working disability, education, professional, retraining

## Abstract

**Objectives:**

We investigate the impact of occupational skin disease consultations among outpatients at the Dermatological Department, University Hospital, Northern Norway.

**Study design:**

From 1997 until 2004, 386 patients with occupational skin disease were examined and given advice on skin care, skin disease treatment, skin protection in further work, and on the legal rights of patients with this disease. Ten to fifteen years later, we wanted to look at these patients in terms of their work situation, the current status of their disease, the help they received from the labour offices, and their subjective quality of life.

**Material and methods:**

In the autumn of 2011 until the spring of 2012, a number of the patients examined in the period from 1997 to 2004 were selected and sent a questionnaire, which they were asked to answer and return, regarding their work situation and the progress and current status of their occupational disease.

**Results:**

A total of 153 (77%) patients answered the questionnaire; 71% of these patients were still in work, and further 15% had old-age retired, 13% were working until then; 16% had retired early because of disability; 54% had changed jobs because of their occupational skin disease; 86% of the patients indicated that the skin disease had improved since our previous investigation.

**Conclusions:**

Our investigation into patients with occupational skin disease documented that the majority of patients who had received professional dermatological consultation and intervention offers were still in the labour market and had good control of their skin disease 10–15 years later. We discovered that 71% of the patients were still employed. 13% had remained in work until they became old age pensioners. Only 16% dropped out of work because of disability. These high percentages may indicate that our intervention has contributed positively to patients’ work conditions and the course of their skin disease.

Contact dermatitis is common in the population of Northern Europe (1,2), and more than 90% of cases are that of hand eczema. A distinction is made between irritant contact dermatitis (ICD) and allergic contact dermatitis (ACD). The consequences of contact dermatitis are discussed in several studies (3,4) because it is the most frequent occupational disease and results in significant costs in terms of treatment, sick leave due to disability at work, extensive retraining, and in-service education. Retraining and further education measures are intended to help patients into more suitable work, taking into account their skin disease, which as a rule means work in a clean and dry environment. Because of the expenses arising from occupational skin disease, insurance companies focus extensively on these disabling diseases (5). Annual costs for patients with occupational hand eczema are high, similar to those with severe psoriasis and severe atopic dermatitis (6).

Several studies conclude that occupational skin diseases result in a reduced quality of life for patients (7,8), and a number of studies show that intervention provides socio-economic benefits, improving both the state of the patients’ skin disease and their quality of life (9), as measured by the Dermatological Life Quality Index (DLQI). Investigations have shown that intervention measures given to these patients are cost-effective (10). Authors generally focused on the effect of intervention on dermatitis and quality of life; in the literature we included, the focus was not on individual professions, except for one article on hairdressers (11).

Our investigation reports on the state of patients’ skin diseases and work situations 10–15 years after the initial consultations. The study was carried out by a professional dermatologist and was medically documented (Appendix 1). Patients were given general advice in the form of written guidelines and also particular advice specific to their profession. We used a questionnaire (Appendix 2) to highlight the patients’ situation, their subjective quality of life, how many stayed in work, how many changed jobs, and how many needed retraining to change to more suitable work. We also wanted to examine whether it is likely that our intervention, so many years ago, had had an impact on the patients’ situation by the time of the questionnaire. Our investigation is the first one in Norway on this subject. The National Research Institution for Occupational Environment and Occupational Health (STAMI) has stated that work-related skin diseases in Norway may, to a great extent, be underreported (12). STAMI has started to draw attention to this issue, and several publications are due in the near future.

## Material and methods

Between 1997 and 2004, 386 patients (40% men, 60% women) were registered and assessed by the author, R. Braun, at the Department of Dermatology for suspected occupational skin disease: age<20 years (2%); 20 to 39 years (58%); 40 to 59 years (37%); >60 years (3%).

The diagnoses were as follows: 53% had ICD, 23% of ICD occurred in combination with contact allergy, and 10% had ACD only. Two percent had a worsening, generalized atopic eczema, and 12% had other occupational diseases. Patients came from 11 occupational groups, mostly from industry with 28%, followed by health care workers 17%, food workers 15%, hair dressers/cosmeticians 9%, office workers 8%, cleaners 6%, shop and pay office workers 6%, fish industry 5%, farmers 3%, flower workers and gardeners 1.5%, housewives 1.5%.

From 2011 to 2012, every second patient was then selected from these 386 patients, in total 198 patients (44% men, 56% woman; age<20 years (4%), 40 to 59 years (41%), >60 years (2%)). They received a letter with a questionnaire developed by the authors (Appendix 2), which they were asked to answer and return. A hospital secretary was the contact to whom the questionnaires were sent and from whom the answers were retrieved; the questionnaires were number coded for delivery to the authors.

The main questions asked were whether the patients were still in work, had changed jobs because of their skin disease, had been retrained, and were satisfied with the consultation and information given at the Department of Dermatology. In addition, patients were asked to describe the current state of their skin disease compared to the earlier condition at the consultation. Finally, patients were asked to add their own comments.

The anamnesis form developed by the authors (Appendix 1) was used for consultation with all patients and the outcome then medically documented. One follow-up consultation was done, including the European Standard Patch test and, when indicated, a patch test of the patient's own material.

Patient care at the end of the consultation included information about skin care, possible aggravating factors, protection at work and during leisure time; they also received a form letter describing easy-to-implement protective measures and advice on handling eczema. They were informed of their legal rights regarding occupational skin disease, and about the help available from the social security office (NAV). This was intended to improve or change patients’ working conditions according to the level of their skin damage, and to improve skin care. Patients were also given a treatment regime. In Norway, follow-up care is the responsibility of the patient's general practitioner and NAV.

The aim of the questionnaire was to use our preliminary results as a baseline for further studies with our patient material. Our consultation and intervention regime has not been published previously. The investigation was conducted at the Department of Dermatology, University Hospital, Northern Norway. Results are presented in Tables I–V

Definitions:

*Quality of life*: patients’ subjective responses when asked about their well-being according to skin condition and work situations.

*Sick leave*: time off from work to address health and safety needs (for instance, occupational skin disease) because of work disability for a period of time, without losing pay.

*Working disability*: not being able to perform work because of health (for instance, occupational skin disease).

*Re-education*: additional education, for instance, new education to obtain work that is appropriate, given the occurrence of an occupational skin disease.

*Social Security Office (NAV) role and responsibility*: To assess and assort skin disease as occupational, document sick leave, provide economic support under rehabilitation, help arrange rehabilitation; and access feasible workplaces and education that matches individuals with suitable work, given their skin disease.

**Table I:** Outcome of patients satisfaction with consultation and advice from occupational dermatologist (n=153)

	n	%
Satisfied with consultation and investigation	136	89
Not satisfied	11	7
Not answered	6	4
Consultation helpful	104	68
Not helpful	44	29
Not answered	5	3

## Results

A total of 153 patients (77%) completed the questionnaire, 89% reporting satisfaction with the previous consultation, investigation, and information. Furthermore, 68% of the patients reported benefits from the consultation/information (Table I); 86% of patients reported subsequent improvement or remission of the skin disease (Table II). In addition, several of the patients with unchanged skin disease specified they had learned to cope better with the disease; 61% of patients reported intermittent sick leave due to their skin disease prior to our consultation, but sick leave was reduced afterwards. The majority of patients (71%) were still working 10–15 years after the consultation. An additional 15% of patients were working until they reached the age of retirement, except three patients (Table III). Only 16% of the patients had been permanently disabled, 2% of whom worked 30 to 50% of the time (Table III); 54% of the working population had changed jobs because of an occupational skin disease.

**Table II:** Outcome of patients’ occupational skin disease after our consultations from 1994 to 2004

	n	%
Better	132	86
Unchanged	10	7
Worse	6	4
Not answered	5	3

**Table III:** Numbers of patients in work, with disability, old-age pensioners, those shift work according to questionnaire 10–15 years after consultation (n = 153)

	n	%
Working	109	71
Not working	44	29
Disabled^a^	24	16
Old-age pensioners^b^	23	15
Shift work	82	54
No shift work	50	40
Not answered	9	6

a Three patients worked 30–50%.

b Three patients (2%) were disabled before they became old-age pensioners.

About 55% of patients had received official confirmation that their skin disease was an occupational disease (Table IV). The self-employed had, as a rule, no insurance for occupational diseases and saw no reason to apply for legal confirmation of their condition; 36% of patients sought retrain, and 33% had their applications granted; 6% of the patients had not completed the retrain for a new profession because they had already received a suitable job offer; 21% applied for and got claim damages. Improvement in the work place was only reported in 27% of cases (Table V).

**Table IV:** Number of patients with occupational dermatitis acknowledged as occupational disease, with grants and rehabilitation from the Social Security Office (NAV) (n = 153 in each group)

	n	%
Approved occupational disease	84	55
Claim damages	32	21
Applied vocational rehabilitation	55	36
Granted vocational rehabilitation	50	33
New education under vocational rehabilitation	41	27

**Table V:** Number of patients reporting improvement in workplace after consultation by dermatologist (n = 153)

	n	%
Improvement in workplace	41	27
No improvement	103	67
Not answered	9	6

About 68% of patients spontaneously submitted comments in the questionnaire (Appendix 3). Of these, 59 patients (57%) expressed their appreciation about being taken seriously during the investigation, said that they had been given information and understanding of their occupation-related disease, as well as useful information about skin care, aggravating impacts, and protective measures that were easy to implement. These factors were crucial to managing continuing investigations. This included clarification on possible causal connections between work and their occupational skin disease, which was highlighted as important. In addition, encouragement, support, and advice in connection with changing profession were central. It was also important to patients to have the care of a qualified dermatologist (13); 22% of patients called for follow-up sessions in the 10–15 years after the first consultations with a dermatologist, and stressed that they had had no continuity with their general practitioners. They felt alone in the restructuring process. Seven patients (7%) expressed a desire for better supervision at the NAV, and more information on rights. Nine (9%) patients found it difficult to remember certain factors so long ago.

## Discussion

Nearly 3 out of 4 patients with an occupational disease were still working, according to the questionnaire, and 54% had changed jobs. Another 15% were gainfully employed until they retired, except three patients (Table III). Only one in 6 patients received disability benefits due to the occupational skin disease. Our results are consistent with other studies (6,9). Reports from other countries describe intervention as a significant factor in achieving improved quality of life and reduced costs (13). In-patient intervention seems to give better results than out-patient intervention.

STAMI reported that 30% of occupational diseases in Norway are skin diseases (12). A table in their article lists those groups that have most occupational skin diseases. The leading group was mechanics, then health care workers, hairdressers, building industry workers, food workers, road workers, farmers, chemical industry workers, and cleaners. The article identifies the main exposure factors as water; cleaning products; oil; and dry, inside air, and then goes into more detail about cleaning products, other chemical substances, oils, fuels, solvents, metals, adhesive and epoxy substances, plastic and rubber products, dust, fibres and minerals, cement, plants, and cosmetics. The same article assumes widespread underreporting of the problems, and therefore the data about occupations and exposure factors are unreliable. STAMI is now reprocessing data from the last few decades that should show how many patients were assessed with occupational skin disease; the results should be available in 3 years’ time (personal communication from Y. Samant to the author). Other European literature sources discuss eczema, quality of life as assessed by DLQI, and the cost-effectiveness of interventions, such as providing information, learning treatment processes, and avoiding exposure factors. Most authors do not provide information on the occupations of patients; we found 1 article specifically on hairdressers but no data for other high-risk occupations. The high number of our patients still in work 10–15 years later is in line with data on interventional patients in this article (11), which implies that we have made a contribution with our outpatient consultation and intervention strategies. Also in the comments, more than half of the patients stress the importance of professional information and support by a dermatologist.

NAV has recently stated publicly that they want to increase their efforts for patients who are at risk of dropping out of the labour market. In this target group, more than 30% are patients with occupational skin diseases. Our contributions to dermatology patients can be regarded as a significant factor here.

According to the answers in our questionnaire, the majority of patients, 89%, were satisfied with our investigation. They felt that their skin condition was taken seriously and also felt taken care of in a difficult situation.

Two out of 3 patients reported that the consultation and investigation 10–15 years ago had been useful. The one-third of patients who considered it not useful may have unrealistic expectations that are beyond our capabilities to fulfil, such as workplace changes by employers, and follow up by NAV and the Labour Inspection. Patients have not always had satisfying follow-up treatment from their general practitioners after their consultation at the Dermatology Department. We think it is crucial to continue proving the information given by dermatologists.

Although some patients gave the answer “not useful” in the questionnaire, they emphasized in a separate comment that it was useful to have been diagnosed with a proven allergy, to have obtained information about toxic substances on the skin during work, and to have been made aware of other preventive measures. “Usefulness,” accordingly, appears higher than shown in Table I.

That occupational skin diseases constitute a large group of occupational diseases are also shown in studies from Sweden and Finland, they account for more than 25% of all occupational diseases (14,15). Hand eczema is the most common occupational skin disease, resulting in significantly reduced quality of life (16–18) and often long sick leave and loss of employment (19). Not infrequently, there are subsequent secondary psychological problems such as depression and anxiety (20,21). According to published studies, occupational intervention, during which sufficient information is given, may increase the patient's quality of life (22,23). Intervention has been shown to prevent flare-ups and worsening of the disease (9,13). Occupational skin diseases entail significant economic costs (24,25). Intervention not only increases patient quality of life but also reduces the economic costs in terms of reduced costs for health care and reduced sick leave (26). Several investigations show that such intervention is cost-effective (27,28). A German study showed as much as 62.9% patients on sick leave before they received any kind of intervention (9).

In a retrospective intervention study of hairdressers, 71.8% of the intervention group was employed, but only 60% in the control group (11). Increased expenses due to occupational diseases are also a critical issue for insurance companies (5,29,30). The dermatological dissemination of knowledge about skin care and treatment, as well as information about the causal relationship between influences at work and skin disease, is important in order to encourage the patient to carry out the tasks (31).

Cumulative exposure to toxic substances is a leading cause of occupational skin disease (1,4). There is usually not just one single cause, but the synergistic effects of various factors leading to the development of occupational dermatitis. The patients may not be in a position to recognize these factors as risk elements. Reducing the impact of a work environment that contributes to the development of eczema requires insight into these factors, and knowledge of how they can be prevented by simply replacing products, or changing jobs (2,3). Sometimes no intervention will help, while in some workplaces no intervention is feasible. In the latter case, the patient has to aim for another profession, which does not harm the skin in the same way. Guidance by the dermatologist can help the patient not to make the same mistake by taking a new job with the same risk of skin disease.

Patients also need information about their rights regarding occupational diseases and vocational rehabilitation. When changing jobs, it may sometimes be sufficient to take certain courses, as well as further education. Experience in the Netherlands and Germany shows that occupational intervention and advice are useful for patients’ employment, and thus the patient's quality of life. This again has socioeconomic consequences (6,26).

To sum up, all these reports indicate that it is worth spending resources on occupational intervention in order to avoid higher financial costs and improve the quality of life of patients.

According to the answers in our questionnaire, neither employers nor the NAV and Labour Inspection in Norway are sufficiently involved in the process when patients first develop occupational skin disease. One reason may be that doctors rarely send the form for suspected occupational skin disease to the Labour Inspection, despite a legal requirement to do so. Improvements were only made in one out of 4 workplaces. The Labour Inspection is in a position to use its influence to improve the workplace more often. Pointing out possible harmful influences at work early allows faster clarification of conditions, which can then be improved. It can thus contribute to faster changes in employment or retraining.

A large majority of patients had little or no eczema when they answered our questionnaire. Their condition had improved and they had suffered less by following the information guide lines received 10–15 years previously on ways to deal with eczema, the suitability of the kind of work they did, and preventive skin protection and skin care measures.

## Conclusion

The majority of patients in this investigation are young people who suffer from occupational skin diseases but who have many years of productive employment ahead of them. It is therefore important that those who fall completely or partially out of work because of occupational skin disease can rapidly return to employment.

Our investigation results provide evidence that most patients find a solution whether they continue in employment without retraining, or do retraining to change jobs. The high number of patients still in employment 10–15 years after our original occupational dermatology consultation and information programme, compared to intervention studies by other European authors, indicates that even though we do not have a control population, our intervention has shortened patients’ restructuring processes.

Based on responses and comments, we can say that in the years following our intervention, the majority of patients in our investigation have had a better course of disease and less sick leave, and the majority of patients stayed in work 10–15 years after the original consultation for occupational skin disease.

In other words, our consultation and information dissemination at the Department of Dermatology may have contributed to helping the majority of patients improve control of their skin disease, to manage to continue in their occupation or to find new, more suitable employment.

## Appendix 1

Anamnesis Template Occupational-Environmental Skin Disease

Social

Family relationships, housing, pets, leisure, private cosmetic and skin cleaning products

Inheritance

Family diseases with focus on skin diseases/atopy

Patient's diseases now and previously, with focus on skin diseases and atopy

Drugs

Allergies

Schooling/employment

Chronological record of education and employment and short description of work place, tasks, skin problems, skin parts charged by chemicals, irritants including cleaning products and skin care products at work, physical influence.

Employers from all work places

Current skin disease

Chronology e.g. debut, duration, course, relation to work previous examinations and treatment

Skin status at day of examination

(e.g. general condition)

Assessment

Measures/intervention/information

## Appendix 2

Questionnaire about consultation/investigation/information on occupational skin disease 10–15 years ago, and skin disease and work status now

**Table:**

Age today □□	Age at consultation □□	Male □	Female □		
1. Work/Occupation					
a. Are you currently employed?				yes □	no □
b. Same work as before?				yes □	no □
If not: previous work?					
c. New work?				yes □	no □
If yes: what kind of work?					
d. Change of workplace/employer?				yes □	no □
e. Days of sick leave per year because of skin disease					□□□
2. Modifications to the workplace					
Did the employer make changes in the work place?				yes □	no □
3. Disability insurance, pensioner insurance					
a. Do you receive disability insurance? %?				yes □	no □
Because of occupational skin disease?					
Any other reasons?					
b. Do you get a pension plan?				yes □	no □
4. Labour Exchange					
a. Was the Labour Exchange involved?				yes □	no □
b. Has the Labour Exchange ordered modifications to workplace?				yes □	no □
5. Your skin disease					
a. Does your skin disease cause you less distress today?				yes □	no □
b. Skin disease worse today?				yes □	no □
c. Skin disease unchanged today?				yes □	no □
6. Your skin disease examined at Department of Dermatology in relation to your work					
a. Diagnosis in our consultation?					
b. Was your skin disease evaluated as occupational disease?				yes □	no □
c. Did you receive claim damages?				yes □	no □
d. Have you applied for vocational rehabilitation?				yes □	no □
If yes, was this approved?				yes □	no □
e. Did you receive further education in vocational rehabilitation?				yes □	no □
If not, why?					
f. Education, courses, re-training by employee?				yes □	no □
7. Assessment of your skin disease					
a. Assessed in addition at Occupational Disease Department, UNN?				yes □	no □
b. Was assessment by occupational dermatologist useful for your further work life?				yes □	no □
8. Are you satisfied with assessment at the University Hospital, Northern Norway?				yes □	no □
What were you satisfied, not satisfied with?					
Patients were asked to comment on all questions.					
At the end of the questionnaire, patients were asked to give additional comments.					

## Appendix 3

Patients’ comments

104 patients out of 153 (68%) wrote additional comments on the questionnaire.

Of these

59 (57%) patients commented that they had been taken seriously, had a good discussion with the examining dermatologist, the examination was thorough, the advice and treatment offered was good and helpful.

23 (22%) patients wanted to attend further consultations with an occupational dermatologist after the second consultation.

9 (9%) patients were hesitant to answer the questionnaire because they could not remember everything from consultation.

7 (7%) patients said that they were not well informed by NAV about their rights as regards changing jobs, re-education.

3 (3%) patients wrote that they had to wait too long for a consultation (often several months, author's comment).

3 (3%) patients felt that the cause of their skin disease was not clear (these were patients with cumulative irritation dermatitis: authors comment).

2 (2%) patients claimed that their employer did not show any interest in patients’ work-related skin disease.

1 (1%) patient would like us to visit the workplace.

1 (1%) patient wanted the dermatologist to provide information about patient's work-related skin disease to family and employer.
